# Correction: MiR-506 Is Down-Regulated in Clear Cell Renal Cell Carcinoma and Inhibits Cell Growth and Metastasis via Targeting FLOT1

**DOI:** 10.1371/journal.pone.0129404

**Published:** 2015-05-26

**Authors:** Feng-qiang Yang, Hai-min Zhang, Shao-Jun Chen, Yang Yan, Jun-hua Zheng

The second author’s name is spelled incorrectly. The correct name is: Hai-Min Zhang. The correct citation is: Yang F-q, Zhang H-m, Chen S-J, Yan Y, Zheng J-h (2015) MiR-506 Is Down-Regulated in Clear Cell Renal Cell Carcinoma and Inhibits Cell Growth and Metastasis via Targeting FLOT1. PLoS ONE 10(3): e0120258. doi:10.1371/journal.pone.0120258
